# Want to be fit? Start with your mind! The role of the placebo effect in physical fitness in children: a preliminary systematic review and meta-analysis

**DOI:** 10.1038/s41366-023-01413-2

**Published:** 2023-12-11

**Authors:** Magdalena Żegleń, Łukasz Kryst, Przemysław Bąbel

**Affiliations:** 1 Jagiellonian University, Institute of Psychology, Pain Research Group, Kraków, Poland; 2 University of Physical Education in Kraków, Faculty of Physical Education and Sport, Department of Anthropology, Kraków, Poland

**Keywords:** Weight management, Obesity, Paediatrics, Lifestyle modification

## Abstract

Physical activity is crucial to prevent and reduce excess body mass. The placebo effect can influence the outcomes of fitness-related interventions; however, this topic has not yet been extensively investigated in children. Summarising the data on placebo effects in fitness-related interventions is essential to understand this problem better. A systematic review of PubMed, Cochrane, PsycINFO, PsycARTICLES, TripDatabase and Embase was carried out. A meta-analysis of the results of studies with comparable research plans was performed. There were significant differences, favouring the placebo intervention. At the final follow-up, the children in placebo groups had higher maximal heart rates, shorter recovery times, longer ergometry phases, running time and lower peak and average perceived exertion than the control. The placebo effect is present in fitness-related parameters in children, regardless of the Body Mass Index status. It is crucial, as for youth with excess body mass, it is difficult to be active, especially to show appropriate levels of motivation and involvement. Importantly, the benefits of the placebo were the strongest in the motivation/ engagement-related parameters and self-assessed exertion. Notably, the nocebo effect was not observed, which is advantageous when considering placebo interventions in practice.

## Introduction

Childhood overweight and obesity, as well as unfavourable body composition, have become a widespread public health problem, especially in recent decades [1–4].

Physical activity is a crucial way to prevent excess body mass in children and to reduce it if it is already present. Previous research revealed inverse cross-sectional associations between the objective level of physical activity and adiposity, as well as cardio-metabolic risk factors in youth [5–7]. It should also be stressed that fitness in childhood and adolescence has been shown to be one of the determinants of health later in life, which further proves the importance of obtaining an optimal level of it early in life. For instance, the results of a systematic review and meta-analysis of longitudinal studies on this topic revealed a prospective negative relationship between both muscular fitness in childhood/adolescence and adiposity as well as cardiometabolic parameters in later life. Importantly, in the same study, a positive association between fitness and bone health was shown [8].

Considering the benefits of optimal fitness described above, it is crucial to appropriately motivate young people to engage in physical activity. Unfortunately, it is particularly difficult in youth suffering from overweight and obesity. These groups often are also victims of weight stigmatisation, which, contrary to popular societal belief, does not motivate individuals to lose weight. On the contrary, shaming or bullying a person because of excess body weight contributes not only to binge eating, social isolation, and avoidance of health care services, but also decreased physical activity [9, 10]. Taking the above into consideration, it is crucial to develop interventions and approaches that facilitate children’s participation in physical activity, while paying particular attention to those with excess body mass.

The concept of placebo is fundamentally understood as an intervention capable of influencing the organism’s functioning despite lacking the inherent potential to produce such effects [11]. Placebo interventions encompass a diverse range of forms, including medically connoted interventions such as pills, injections, creams, and even sham surgical procedures. However, their scope extends beyond these medical connotations, such as colours or shapes [12–17]. The pivotal determinant of the placebo’s efficacy resides in the contextual elements that accompany its administration, such as the colour, shape, or taste of the placebo pill. Moreover, these same contextual elements exert an influence on active interventions, thereby modifying their effectiveness [11]. The term “placebo effect” denotes the positive physiological and psychological changes resulting from placebo application, exemplified by symptom improvement, such as a reduction in pain intensity [18]. Therefore, it is represented as the difference between placebo and non-treatment/natural history groups [19, 20] When no control group for the placebo group is included in a study design, any changes in the placebo group can be called the placebo response. The placebo response is defined as all health changes resulting from the inactive treatment, including regression towards the mean and the natural course of the disease, among other factors that may be responsible for the changes in the placebo group [19, 20]. Thus, only the placebo effect represents the true effect of the placebo intervention [21].

Analogous to the placebo effect, relatively worse outcomes or adverse effects occurring due to the psychosocial context that cannot be attributed to the active treatment are referred to as the nocebo effect. This can be observed as differences between nocebo and non-treatment/natural history groups and has been described, for instance, in the context of adverse drug events as well as pain-related research [19, 22–25]. The nocebo response is defined as any unfavourable symptom or group of symptoms that the patient experiences in the absence of active treatment.

In recent decades there has been a significant rise in scientific interest regarding the placebo effect in a broad spectrum of contexts and applications [26]. This includes studies looking into the placebo effect in the management of overweight and obesity, as well as in physical activity and fitness. Placebo interventions used in this context range from sham supplements to false information regarding the caloric values of meals or energy expenditure during everyday exercise. Findings of such studies show that placebo interventions can be effective in reducing excess body weight and even the percentage of body fat [27–30]. On the other hand, some findings suggest the presence of the nocebo effect with the use of pharmacologically connoted interventions (e.g., sham weight-loss tablets), which could be a result of the expectations that participants have towards the supplement [31].

Considering all the above, the placebo and nocebo effects could influence the outcomes of fitness-related interventions. Moreover, it should be stressed that these issues have not yet been well described and that no systematic review or meta-analysis shows them in detail. Consequently, summarising the available data on placebo effects in fitness-related interventions is essential to better understand this problem.

A systematic review and meta-analysis of studies concerning the role of the placebo and nocebo effects in physical fitness in the paediatric population were conducted to answer the following questions:

1. Is the placebo effect present in regards to physical fitness test results in the paediatric population?
2. What is the magnitude of the placebo effect in regards to physical fitness test results in the paediatric population?
3. Is the nocebo effect present in regards to physical fitness test results in the paediatric population?
4. What is the magnitude of the nocebo effect in regards to physical fitness test results in the paediatric population?
5. Is there a difference in the magnitude of placebo and nocebo effects in regards to physical fitness test results in the paediatric population?

## Material and methods

The review was conducted according to a previously created and registered protocol (PROSPERO registration no. CRD42022342646; registration date: 9.07.2022). The review process followed the Preferred Reporting Items for Systematic Review and Meta-Analyses Protocols (PRISMA-P checklist form) guidelines and the recommendations on data searching and processing described in the Cochrane Handbook for Systematic Reviews [32–34].

### Searches and included studies

Selected databases (PubMed, Cochrane, Embase, PsycINFO, PsycARTICLES, TripDatabase) were searched for relevant articles. Additionally, the reference lists of the included articles were manually checked for other relevant studies.

Studies with at least two groups (a placebo group and a relevant control group, i.e., no-treatment or natural history group), published in English and focused on the paediatric population (below the age of 18) participating in fitness programs or studies investigating the problem of the placebo/nocebo effect in physical fitness were included in the review and considered for meta-analysis.

Reviews, meta-analyses, secondary publications not based on a systematic review, letters to the editor, and conference communications were not included.

Detailed inclusion and exclusion criteria are presented in Table 1. Full search strategy (Table S1) and a list of excluded articles are provided in the supplementary material.

**Table 1:** Inclusion/exclusion criteria for systematic review.

PICOS item	Inclusion criteria	Exclusion criteria
Population	Paediatric population ( < 18 years of age)	Adults, animal models
Intervention	Fitness programs or studies investigating the problem of the placebo/nocebo effect in physical fitness	Placebo-controlled trials investigating pharmaceutical interventions for physical fitness with no natural history/ no-intervention group
Comparator	The intervention used in the control group (e.g., no-treatment or natural history group)	N.A.
Outcome	Measurements of physical fitness including but not limited to: maximal oxygen uptake (VO_2_max), heart rate (initial and peak), rate of perceived exertion (RPE), recovery time, and results of motor/ physical fitness tests. Measurements presented as absolute values and as change from the initial value will be included.	N.A.
Study	Studies with at least two groups: a placebo group and a relevant control group (e.g., no-treatment or natural history group)	Studies without control groups, review articles, meta-analyses, secondary publications not based on a systematic review, letters to the editor, and conference communications

*N.A.* Not applicable.

### Study selection and data extraction

The study selection was performed in two stages. The first stage included screening the titles and abstracts of articles/publications identified as potentially relevant. This was based on the inclusion/exclusion criteria and was carried out by two independent researchers (M.Z., L.K.). The full texts of potentially relevant studies were reviewed using the same inclusion/exclusion criteria by the same two researchers (M.Z., L.K.). In the case of disagreement which could not be resolved by discussion, a third researcher was consulted (P.B.).

A standardised, pilot-tested form was used for data extraction, independently performed by two researchers (M.Z., L.K.). If consensus was not reached, a third researcher (P.B.) assisted in making the final decision. The following data were extracted from publications:

1. Sample size;
2. Age;
3. Sex;
4. Description of the study intervention (methods, how the placebo effect was elicited etc.);
5. Duration of the study intervention;
6. Methods of assessing physical fitness;
7. Physical fitness indicators before the study intervention;
8. Physical fitness indicators after the study intervention;
9. Methods of assessing the subjective level or change in physical fitness;
10. Subjective satisfaction level or change in physical fitness.

### Risk of bias assessment

Two independent researchers (M.Z. and L.K.) assessed the quality of the included studies. The Cochrane Risk of Bias (RoB) Tool for randomised studies was used to identify additional sources of bias and appraise specific domains. These domains include random sequence generation, allocation concealment, blinding of participants, blinding of outcome assessment, incomplete outcome data, and selective outcome reporting [32, 33].

### Data synthesis

All studies meeting the inclusion criteria were included in a narrative synthesis. Additionally, some studies satisfying the following criteria were included in the quantitative synthesis:

1. Studies using compatible outcome measures;
2. Studies using a similar methodology;
3. Studies in which the data from the control (i.e., no-treatment, natural history) group are available.

Standardised mean difference (SMD) with 95% confidence intervals (CIs) between placebo and control groups were used for continuous outcomes. The random-effects model was used as it is characterised by the greatest generalisability for empirical examination of summary effect measures in meta-analyses [32, 33, 35]. A *P*-value of less than 0.05 was established as the threshold for statistical significance. Review Manager v.5.4 (Copenhagen: The Nordic Cochrane Centre, The Cochrane Collaboration, 2020) and Microsoft Excel^®^ 2019 were used for all analyses performed in the study.

## Results

**Fig. 1:**
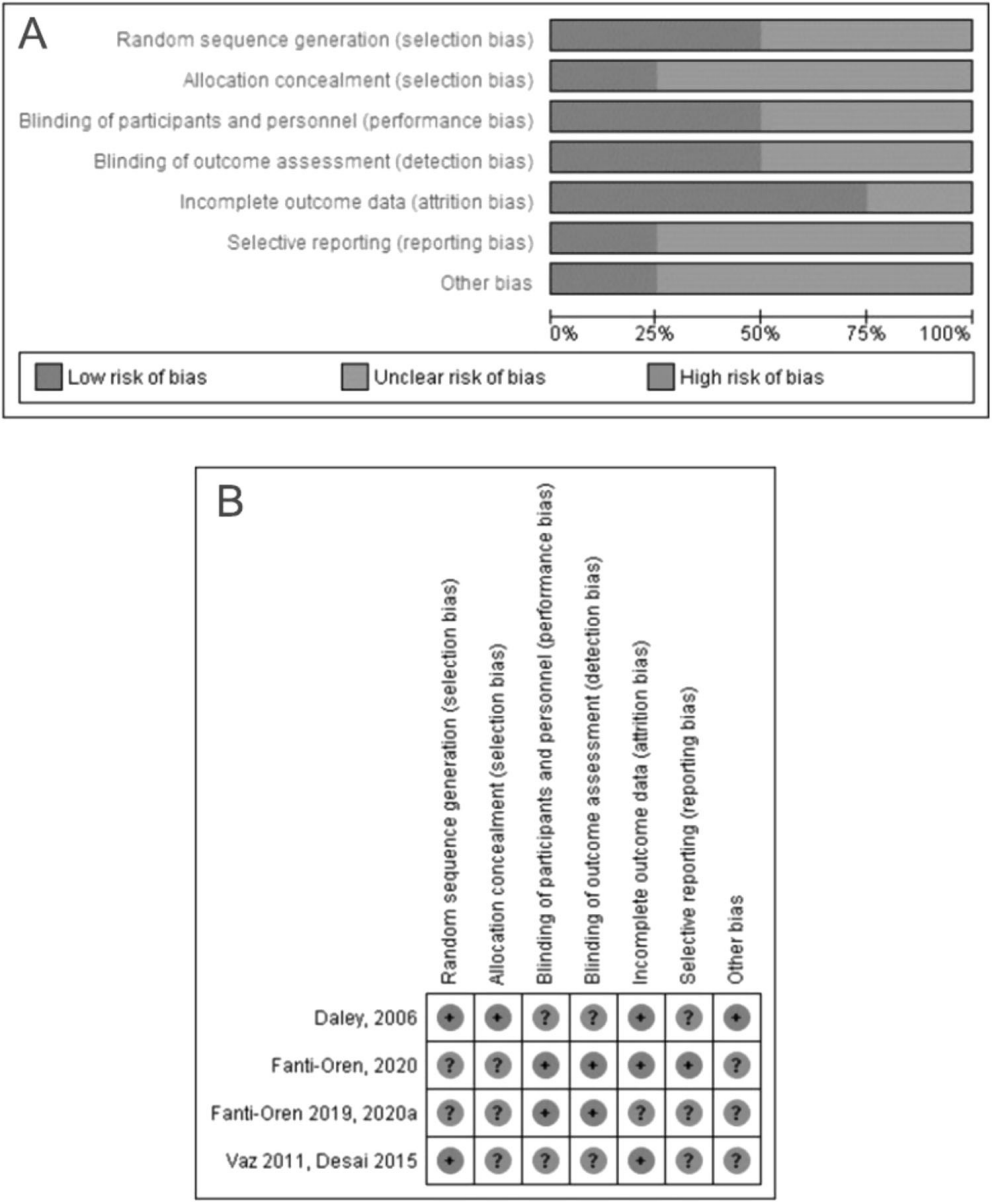
Risk of bias graph and summary and details. **A** presents summarised risk of bias for all of the included studies, while **B** shows detailed scores for each of them.

Four studies described in seven references were included in the narrative review [36–42]. Two of these studies were combined in the meta-analysis [39–41]; the other two studies were excluded from the meta-analysis due to significant methodological differences, mainly study duration (examination immediately after the intervention vs a few months after the intervention) and control group approach (participants being their own control vs standard parallel group design) (Fig. 1).

The study flow diagram is presented in the supplementary material (Fig. S1).

The essential characteristics of the included studies, together with the final follow-up values of the endpoints of interest, are presented in Table 2. All studies were described as randomised or controlled, and three of them were double-blinded. The number of individuals included in each study ranged from 20 to 287; the average age was from 8.18 to 13.1.

**Table 2:** Basic characteristics of the included studies.

Characteristic	Fanti-Oren et al. [41]	Fanti-Oren et al. [39], Fanti-Oren et al. [40]	Vaz et al. [38]; Desai et al. [36]; NCT00876018	Daley et al. [42]
Type of the study	Study described as randomised, controlled, double-blind with parallel-groups	Study described as randomised, controlled, double-blind with parallel-groups	Study described as randomised (block randomisation), controlled, double-blind	Study described as randomised, controlled.
Number of individuals	*N* = 20	*N* = 48	*N* = 287 (*n* = 200 in total in placebo and control/ no-intervention group)	*N* = 81 (*n* = 53 in total in placebo and control group)
Age, mean ± SD [years]	10.43 ± 2.09^	Excess body weight (overweight + obesity): 10.35 ± 2.12 Normal body weight: 9.78 ± 1.65	Placebo: 8.18 ± 1.01 Control: 8.29 ± 1.04	13.1 (no data on SD)
Sex	*n* = 7 girls *n* = 13 boys	Excess body weight (overweight + obesity): *n* = 10 girls; *n* = 14 boys; Normal body weight: *n* = 12 girls; *n* = 12 boys	Placebo: *n* = 50 girls, *n* = 50 boys Control: *n* = 50 girls, *n* = 50 boys	No data
BMI status	Children with obesity (BMI ≥ 95 percentile)	Children with normal weight (BMI percentile ≥ 5 and < 85), overweight (BMI percentile ≥ 85 and < 95) and obesity (BMI percentile ≥ 95)	Z-score of 0 to −3 SD for height-for-age and weight-for-age	Adolescents with a BMI that exceeded the 98th percentile for age and sex
Inclusion criteria	-pre-pubertal (Tanner stage 1) children; -children with obesity; -consent of parents/legal guardians	-pre-pubertal (Tanner stage 1) children; -consent of parents/ legal guardians	-written informed consent from the parents/legal guardians and participants	-written, informed consent; -BMI that exceeded the 98th percentile for age and sex according to 1990 United Kingdom reference data
Exclusion criteria	-organic disease; -medications that might interfere with growth, weight control, or exercise tolerance (e.g., corticosteroids, thyroid hormone substitution, and recombinant growth hormone).	-organic disease; -medications that might interfere with growth, weight control, or exercise tolerance (e.g., corticosteroids, thyroid hormone substitution, and recombinant growth hormone).	-severe anemia (Hb 0.80 g × L21), cardiovascular or respiratory disease, physical disability, recent history of serious infections, or surgery or injuries that would impair their ability to perform the study tests; -individuals who were already taking nutritional supplements, if they had participated in a nutrition intervention study in the preceding year, or if they were family members of staff employed at the study site or with the sponsor	-medical conditions that would restrict the ability to be active 3 times per week for 8 weeks; -unwillingness to attend supervised exercise sessions 3 times per week for 8 weeks; -major cognitive or psychiatric impairments; -diagnosis of insulin-dependent diabetes mellitus or oral steroid treatment
Placebo and control intervention	Before each testing session, the participants drank a glass of a drink: -placebo: the drink was described by the examiner as a drink that increases energy levels, strengthens muscles, and is therefore likely to improve exercise performance; the bottles were opaque, blue-coloured, and included a label proclaiming the content to be an energy drink that strengthens muscles and improves athletic performance; -control: the participants were informed that they are drinking water; the bottles were standard, transparent water bottles.	Before each testing session, the participants drank a glass of a drink: -placebo: the drink was described by the examiner as a drink that increases energy levels, strengthens muscles, and is therefore likely to improve exercise performance; the bottles were opaque, blue-coloured and included a label proclaiming the content to be an energy drink that strengthens muscles and improves athletic performance; -control: the participants were informed that they are drinking water; the bottles were standard, transparent water bottles.	Placebo group was administered an unfortified choco-malt beverage powder (energy equivalent of fortified choco-malt powder in experimental group) as single servings of 40 g in 100 mL water. Administration once a day for 4 months under supervision of study personnel on all school days (6 days per week) and for self-consumption at weekends and/ or on holidays. Control group received no intervention.	Placebo: instead of aerobic exercise, participants performed light body-conditioning/stretching exercises, during which HR was maintained at 40% of HR reserve, and no exercise counselling or behavioural change advice was given. Control (usual care): participants were asked to continue with their lives as normal.
Duration of the study	The participants were tested twice – once with water and once with placebo drink, in random order.	The participants were tested twice – once with water and once with placebo drink, in random order.	120 days	After participants completed their 8-week interventions, they were given an individualised home exercise or body conditioning program (in line with their group assignments) to follow on their own for an additional 6 weeks (14-week follow-up period)
End points of interest	-heart rate (initial and maximal), measured using Polar H10 heart rate monitor; - recovery time, measured as the time from the end of exercise until heart rate reached 100 bpm; -rate of perceived exertion (RPE), evaluated using the Borg scale -duration of ergometry phase	-heart rate (initial and maximal), measured using Polar H10 heart rate monitor; -recovery time, measured as the time from the end of exercise until heart rate reached 100 bpm; -rate of perceived exertion (RPE), evaluated using the Borg scale -duration of ergometry phase	-maximal aerobic capacity (VO_2_max) - defined as the maximum rate of oxygen consumption, measured during incremental exercise (externally placed 12-inch step test); -aerobic capacity(VO_2_peak) - defined as maximum rate of oxygen consumption attained on a particular exercise test (20 m shuttle run test); -40 m sprint was used to assess speed with time taken to complete the sprint being recorded manually using a digital stopwatch; -maximal handgrip strength for dominant and non-dominant hand, measured using Jamar hand dynamometer; -time to fatigue, defined as the time in seconds taken for the handgrip to fall from maximal value to 50% of the maximal value; -rate of decline of muscle strength was assessed to measure the muscle endurance of forearm; sustained isometric contraction of forearm flexors to 50% of maximal handgrip was measured using the Jamar hand dynamometer and was performed on the non-dominant arm.	-the poorly fit category of the modified Balke protocol was used to assess fitness (distance walked was recorded as a result); -resting heart rate
Participants lost to follow-up/ who discontinued the study	All of the participants completed the study.	All of the participants completed the study.	13 participants did not complete the study, of whom 2 were lost to follow-up and 11 withdrew consent (including in *n* = 4 placebo and n = 2 in control)	7 participants lost to follow-up (1/23 in placebo and 5/30 in control group).
Baseline values for the endpoints of interest	N.D.	N.D.	Placebo (*N* = 95), mean ± SD: -VO_2_max: 37.9 ± 5.6; -VO_2_peak: 32.8 ± 4.1; -40-meter sprint [s]: 8.9 ± 1.0; -maximal handgrip strength (dominant hand) [kg]: 9.4 ± 3.7; - maximal handgrip strength (non-dominant hand) [kg]: 8.6 ± 3.5; -time to fatigue in handgrip strength test [s]: 12.36 ± 7.66; -decline of muscle strength [kg/s]: 0.38 ± 0.24. Control (N = 97), mean ± SD: -VO_2_max: 37.0 ± 5.3; -VO_2_peak: 32.4 ± 4.2; -40 meter sprint [s]: 8.9 ± 1.0; -maximal handgrip strength (dominant hand) [kg]: 9.8 ± 3.4; -maximal handgrip strength (non-dominant hand) [kg]: 8.5 ± 3.2; -time to fatigue in handgrip strength test [s]: 11.13 ± 9.85; -decline of muscle strength [kg/s]: 0.42 ± 0.26.	Placebo (*N* = 95), mean ± SD: -resting heart rate [bpm]: 82.26 ± 8.30; -aerobic function [miles]: 0.36 ± 0.10; Control (*N* = 95), mean ± SD: -resting heart rate [bpm]: 84.62 ± 10.57; -aerobic function [miles]: 0.38 ± 0.12;
Final follow-up values for the end points of interest	Placebo (*N* = 20), mean ± SD: -initial heart rate (bpm): 104.6 ± 12.8; -ergometry phase: 5.1 ± 1.2*; -running time [s]: 559.9 ± 151.0; -maximal heart rate (bpm): 176.1 ± 13.7*; -peak RPE: 18.0 ± 1.1; -average RPE: 12.1 ± 2.3*; -recovery time (s) 119.2 ± 25.3* Control (*N* = 20), mean ± SD: -initial heart rate (bpm): 104.2 ± 11.6; -ergometry phase: 3.9 ± 1.2; -running time (s): 434.4 ± 140.3; -maximal heart rate (bpm): 167.5 ± 16.8; -peak RPE: 19.1 ± 1.5; -average RPE: 13.6 ± 2.1; -recovery time (s)133.2 ± 23.7	Placebo (*N* = 20), mean ± SD: -excess body weight (obesity+overweight): -initial heart rate (bpm): 103.8 ± 11.8; -ergometry phase: 4.8 ± 1.4*; -running time (s): 521.5 ± 182.5*; -maximal heart rate (bpm): 174.2 ± 14.8*; -peak RPE: 17.9 ± 1.7*; -average RPE: 12.5 ± 2.5*; - ecovery time (s)118.4 ± 31.6*; -normal body weight: -initial heart rate (bpm): 95.0 ± 7.6*; -ergometry phase: 7.6 ± 1.3*; -running time (s): 893.3 ± 150.1*; -maximal heart rate (bpm): 189.8 ± 12.2*; -peak RPE: 16.2 ± 1.5*; -average RPE: 10.7 ± 1.5*; -recovery time (s)96.7 ± 17.8*; Control (*N* = 20), mean ± SD: - excess body weight (obesity+overweight): -initial heart rate (bpm): 103.8 ± 11.8; -ergometry phase: 3.6 ± 1.4; -running time (s): 396.9 ± 161.9; -maximal heart rate (bpm): 165.8 ± 16.7; -peak RPE: 19.2 ± 1.0; -average RPE:14.1 ± 2.5; -recovery time (s)132.2 ± 28.5; -normal body weight: -initial heart rate (bpm): 94.8 ± 7.4; -ergometry phase: 6.0 ± 1.3; -running time (s): 700.1 ± 155.2; -maximal heart rate (bpm): 177.9 ± 13.6; -peak RPE: 18.3 ± 1.4; -average RPE: 12.1 ± 1.4; -recovery time (s)106.7 ± 18.6.	Placebo (*N* = 95), mean ± SD: -VO_2_max: 37.6 ± 5.3; -VO_2_peak: 38.6 ± 3.7; -40 meter sprint [s]: 8.9 ± 1.0; -maximal handgrip strength (dominant hand) [kg]: 10.6 ± 3.0; -maximal handgrip strength (non-dominant hand) [kg]: 9.5 ± 3.0; -time to fatigue in handgrip strength test [s]: 14.62 ± 6.35; -decline of muscle strength [kg/s]: 0.33 ± 0.16. Control (*N* = 97), mean ± SD: -VO_2_max: 37.8 ± 5.3; -VO_2_peak: 38.2 ± 4.2; -40 meter sprint [s]: 8.7 ± 1.0; -maximal handgrip strength (dominant hand) [kg]: 10.9 ± 3.2; - maximal handgrip strength (non-dominant hand) [kg]: 9.6 ± 2.6; -time to fatigue in handgrip strength test [s]: 14.03 ± 7.25; -decline of muscle strength [kg/s]: 0.35 ± 0.16.	Placebo (*N* = 95), mean ± SD: -resting heart rate [bpm]: 80.24 ± 1.01; -aerobic function [miles]: 0.37 ± 0.01; Control (*N* = 95), mean ± SD: -resting heart rate [bpm]: 81.12 ± 1.10; -aerobic function [miles]: 0.36 ± 0.01;

Regarding the Body Mass Index (BMI), the two studies included in the meta-analysis were carried out among children with normal weight (BMI percentile ≥ 5 and < 85), overweight (BMI percentile ≥ 85 and < 95) and obesity (BMI percentile ≥ 95) (three references by Fanti-Oren et al.). Thus, in the primary analysis, the results were pooled for all BMI categories. The two additional studies included in the narrative review concerned children with underweight (Z-score of 0 to −3 SD for height-for-age and weight-for-age [Desai et al.; NCT00876018; Vaz et al.]) and obesity (BMI that exceeded 98th percentile for age and sex [Daley et al.]).

Placebo interventions used in the studies included a drink given to children with an explanation that it strengthens muscles and improves athletic performance (three references by Fanti-Oren et al.), an unfortified choco-malt beverage powder (described as a placebo by the authors of the study [Desai et al.; NCT00876018; Vaz et al.]), and a placebo exercise routine (exercise designed not to elevate heart rate [Daley et al.]).

### Heart rate

**Fig. 2:**
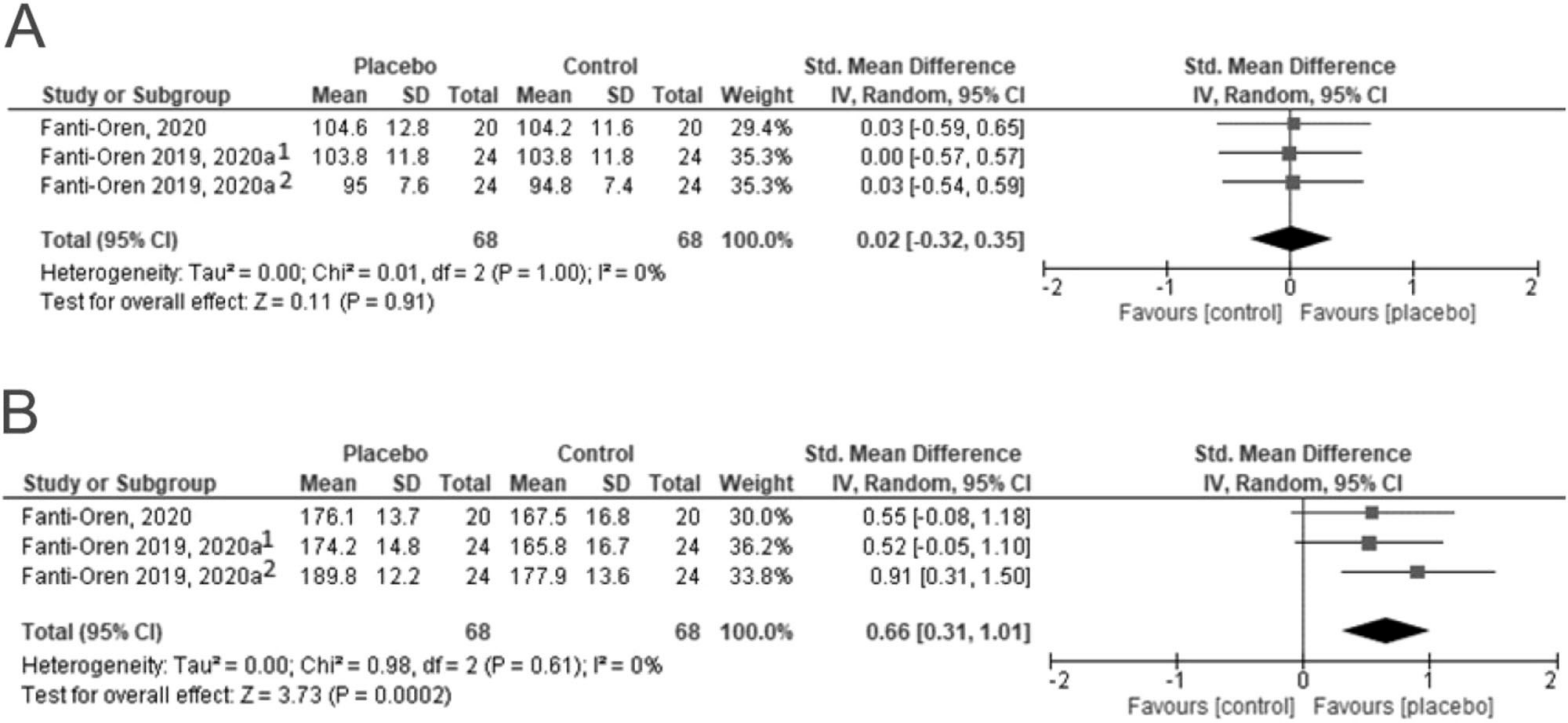
Initial and maximal heart rate [bmp] (^1^excess body weight; ^2^normal body weight). **A** presents individual and pooled results for the assessment of the initial heart rate, while **B** shows the same for the maximal heart rate.

Heart rate was examined in three of the four studies (three references by Fanti-Oren et al. and Daley et al.). Two took into consideration initial (resting) as well as maximal heart rate during exertion (Fanti-Oren et al.), while one analysed only resting heart rate (Daley et al.). All studies showed that the initial heart rate was comparable in the placebo and control groups. Also, the pooled results of the meta-analysed studies did not show significant differences between the groups (SMD = 0.02[−0.32, 0.35]). The maximal heart rate at the final follow-up differed significantly only in the normal-weight children when individual results were considered. However, when the pooled results were analysed, there was a statistically significant difference in favour of the placebo group (SMD = 0.66 [0.31; 1.01]) (Fig. 2).

### Recovery time

**Fig. 3:**
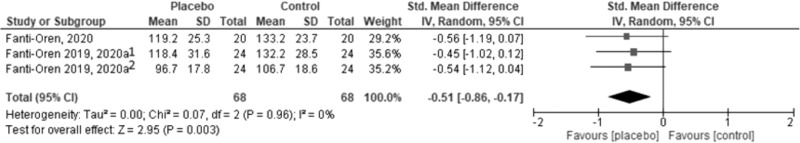
Recovery time [s] (^1^excess body weight; ^2^normal body weight). The figure presents individual and pooled results for the assessment of the recovery time (measured as the time from the end of exercise until heart rate reached 100 bpm).

Recovery time was analysed in two studies in all three of the BMI-based categories (three references by Fanti-Oren et al.). In the meta-analysis, at the final follow-up the individual results were not statistically significant, although there was a trend favouring the placebo each time. Nevertheless, when the results were pooled, children in placebo groups had significantly shorter recovery times than those in control groups (Fig. 3).

### Ergometry phase

**Fig. 4:**
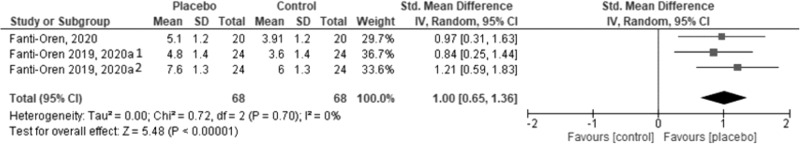
Ergometry phase [s] (^1^excess body weight; ^2^normal body weight). The figure presents individual and pooled results for the assessment of the duration of ergometry phase.

The duration of the ergometry phase was analysed in two studies in all three BMI-based categories (three references by Fanti-Oren et al.). At the final follow-up, the children in placebo groups had significantly longer ergometry phases compared to those in control groups; this was the case for the individual as well as pooled results (SMD = 1.00 [0.65; 1.36]) (Fig. 4).

### Running time

**Fig. 5:**
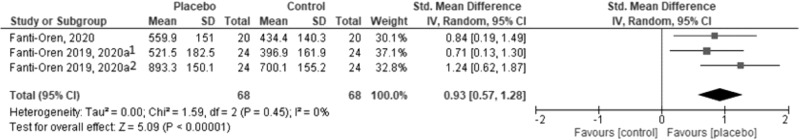
Running time [s] (^1^excess body weight; ^2^normal body weight). The figure presents individual and pooled results for the assessment of the duration of the exercise (running).

Running time was analysed in two studies in all three BMI-based categories (three references by Fanti-Oren et al.). At the final follow-up, the children in placebo groups had significantly longer running time than those in control groups; this was the case for individual and pooled results (SMD = 0.93 [0.57;1.28]) (Fig. 5).

### Rate of perceived exertion

**Fig. 6:**
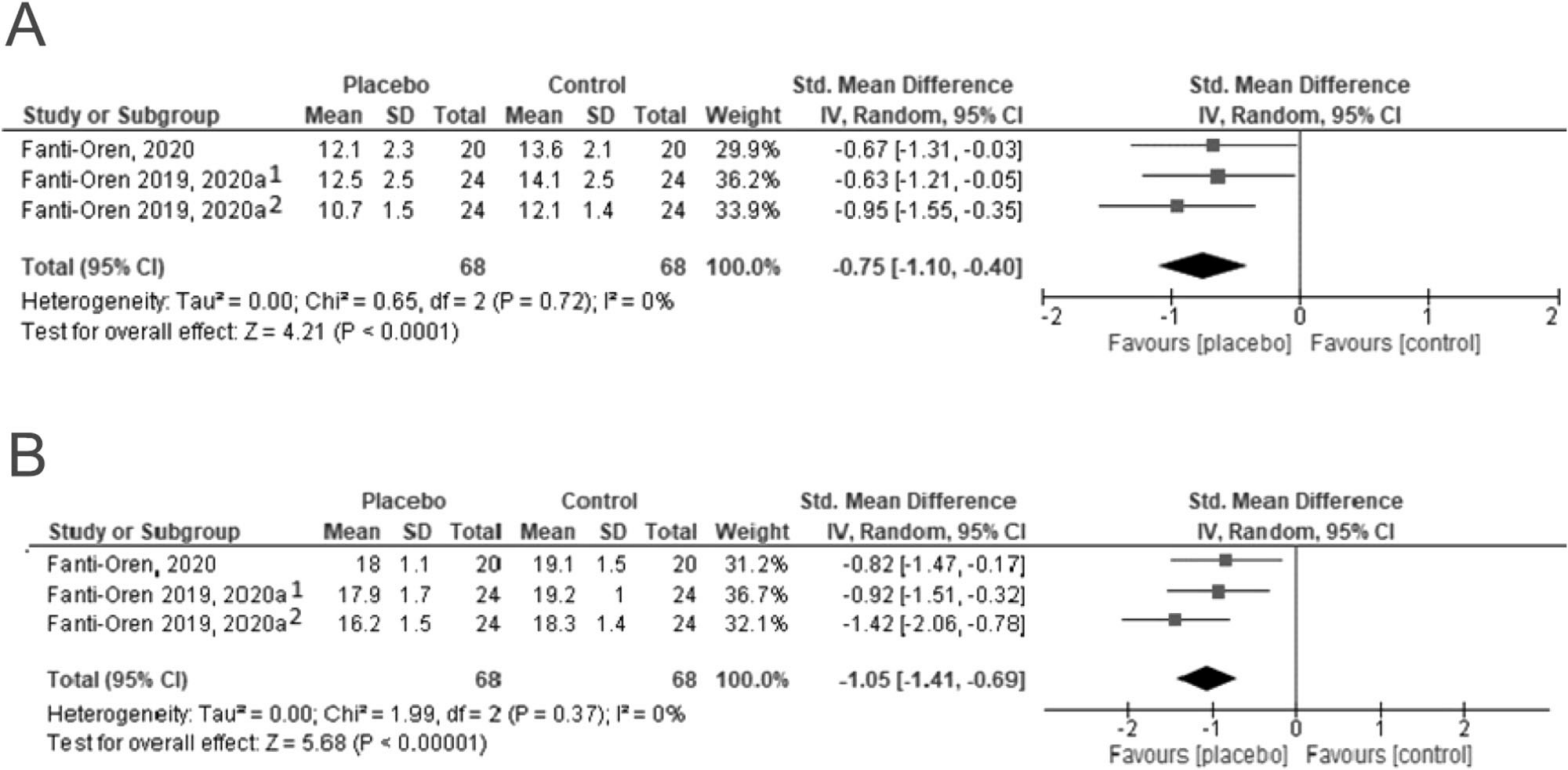
Average and peak perceived exertion (^1^excess body weight; ^2^normal body weight). **A** presents individual and pooled results for the assessment of the average perceived (self-reported) exertion, while **B** shows the same for the peak exertion.

The average and peak rates of perceived exertion were analysed in two studies in all three BMI-based categories (three references by Fanti-Oren et al.). At the final follow-up, the placebo groups had a significantly lower peak and average perceived exertion than the control; this was the case for the individual as well as the pooled results (peak rate of perceived exertion SMD = −1.05 [−1.41;−0.69]; the average rate of perceived exertion SMD = −0.75 [−1.10; −0.40]) (Fig. 6).

### Oxygen consumption (VO2) and aerobic function

One study which was included in the narrative synthesis analysed maximal and peak oxygen consumption (Desai et al.; NCT00876018; Vaz et al.). At the final follow-up, both maximum and peak VO2 values were comparable between the placebo and control groups.

One study that was included only in the narrative synthesis measured aerobic function (Daley et al.). At the final follow-up, this parameter’s value was comparable between the placebo and control groups.

### Handgrip and muscle strength

Maximal handgrip strength (for dominant and non-dominant hands), time to fatigue, and decline of muscle strength in the handgrip strength test were analysed in one study that was included only in the narrative synthesis (Desai et al.; NCT00876018; Vaz et al.). At the final follow-up, maximal handgrip strength was comparable in the placebo and control groups. On the other hand, children in the experimental group had a slightly longer time to fatigue and a slower muscle strength decline than their counterparts who were randomised to the control group.

### 40-meter sprint

Time taken for a 40-meter sprint was analysed in one study that was included only in the narrative synthesis (Desai et al.; NCT00876018; Vaz et al.). Children in the placebo and control groups had comparable test results at the final follow-up.

### Sensitivity analysis

Sensitivity analysis included children with excess body mass. The results were analogous to those obtained in the main analysis.

## Discussion

The systematic review and meta-analysis revealed significant differences in fitness parameters, thus showing that the placebo intervention was effective. Particularly at the final follow-up, the children in placebo groups had higher maximal heart rates, shorter recovery times, longer ergometry phases and running times (duration of running), as well as lower peak and average perceived exertion than the control groups.

The greatest effect size, which indicates a large practical effect, was noted for the peak rate of perceived exertion (SMD = − 1.05 [−1.41; −0.69]). On the other hand, the smallest effect size, which suggests the lowest practical significance, was observed for the initial heart rate (SMD = 0.02 [−0.32; 0.35]). Taking into consideration only the outcomes for which a statistically significant difference was noted between the placebo and control groups, the smallest effect size was observed for recovery time (SMD = −0.51 [−0.86; −0.17], which indicates moderate practical significance.

Based on the results of the meta-analysis, the benefits of the placebo interventions were most visible in the case of parameters related to motivation/engagement in and self-assessment of effort associated with the exercise. On the other hand, there was no significant difference between the groups in regard to physiological features, such as VO_2_ (max and peak), initial heart rate and handgrip strength. The only significant difference in such parameters was noted for maximal heart rate; this was most likely due to extended running duration in children randomised to placebo groups. It should be stressed that even though children in the placebo groups could run for a longer time with a longer ergometry phase, they also reported lower peak exertion and average exertion.

Positive effects of placebo exercise-related interventions on psychological aspects of fitness and well-being have previously been documented in adults. For instance, in a study by Desharnais et al., young adults whose expectations of the psychological benefits of exercise were strengthened by an expectancy modification procedure reported more improved self-esteem compared to control subjects [43]. Some researchers also argue that, in general, the psychological effects of physical activity might be mainly due to the placebo effect associated with exercise alone [27, 44]. Current results do not support this concept: the children randomised to the control groups also participated in the same study programs to which the study group was subjected. Thus, the obtained effects may only be attributed to the placebo intervention.

It should be stressed that the placebo effect observed in the current study was observed regardless of the BMI status, i.e., in children with normal weight, as well as those with excess body weight. This is essential because children with overweight and obesity are often challenging to motivate to participate in physical activity; they frequently drop out or do not exhibit appropriate levels of motivation [45–47]. It has been suggested that uncomfortable body sensations might be essential factors that contribute to the difficulties observed in children with excess body weight regarding exercise [48]. Considering that the use of the placebo in the current study was associated, among others, with lower levels of perceived exertion, such interventions can at least partially counteract the adverse body sensations associated with exercise. Efficient methods of establishing appropriate levels of motivation and positive practices regarding physical activity are also crucial because behaviours and habits established at developmental age tend to be maintained throughout adulthood [45, 49].

Considering the forms of placebo used in the included studies, the sham energy/strength-enhancing drink seems to be the most effective. On the other hand, two other placebo interventions (unfortified cocoa powder and placebo exercise routine) seemed to be less effective. However, it should also be taken into consideration that the studies based on these two interventions did not measure the subjective outcomes, only the objective ones (e.g., VO_2_, handgrip strength etc.).

The nocebo effect was not observed in any of the analysed studies. This means that none of the placebo interventions used in the studies had unfavourable or adverse effects in regard to the analysed outcomes, which is very advantageous when considering the use of placebo interventions in practice.

The results obtained in the current study should be considered with the understanding of certain limitations. Firstly, due to the methodological restrictions of included studies, there is a lack of possibility to control the effects of possible mediating factors, such as age, sex and others (e.g., level of physical activity, sedentary behaviours, diet, etc.). Additionally, the results and conclusions were obtained based on only four studies. On the other hand, no other research regarding the analysed topic that meets the inclusion criteria of the review is currently available. Thus, the conclusions are based on the best available evidence. The use of final values instead of the differences between the baseline and the last follow-up in the meta-analysis could be a limitation. Such an approach was chosen because of the lack of standard deviations between the baseline and the last follow-up in most of the included studies. Extrapolation or estimation of SDs was also not possible due to insufficient data. It should be stressed that the approach utilised in the review follows the recommendations of the Cochrane Collaboration, according to which post-intervention results in randomised trials estimate the same value as the comparison of changes from baseline [50].

In conclusion, the placebo effect is present regarding fitness-related parameters in children. Notably, the placebo effect was observed in all analysed groups of children, regardless of the BMI status. This is crucial because youth with excess body mass are often extremely difficult to motivate to participate in physical activity or, especially, to show appropriate levels of motivation towards exercise. In the context of the last conclusion, it should be stressed that the benefits of the placebo intervention observed in the current study were strongest in the parameters related to motivation and self-assessed exertion.

### Supplementary information

Want to be fit - supplementary material
