# Supplementary material for: Want to be fit? Start with your mind! The role of the placebo effect in physical fitness in children: a preliminary systematic review and meta-analysis
Source: Int J Obes (Lond). 2023 Dec 11;48(2):177–87. doi: 10.1038/s41366-023-01413-2 (PMC10824660; doi:10.1038/s41366-023-01413-2)

Table. S1.The search strategy used in the review.

Query	Keywords/ descriptors	PubMed	Embase	Cochrane
#1.	*"fitness"*[1, 3]<br>*''fitness'/exp OR fitness'*[2]	113 026	127 653	14 069
#2.	*child*[1,2,3]	2 982 577	3 021 168	182 406
#3.	*paediatric OR pediatric*[1,2,3]	1 112 271	1 300 402	57 640
#4.	#2 OR #3	3 329 181	3 534 460	196 330
#5.	*Placebo* [1,2,3]	254 950	505 489	350 859
#6.	*"Placebo effect"* [1,3]<br>*'placebo effect'* [2]	7 478	9 943	4 960
#7.	#5 OR #6	254 950	505 489	350 866
#8.	#1 AND #4 AND #7	45	63	222
#9.	*nocebo* [1,2,3]	1 046	1 504	305
#10.	*"nocebo effect"*[1,3]<br>*'nocebo effect'* [2]	661	1 115	204
#11.	#9 OR #10	1 046	1 504	305
#12.	#1 AND #4 AND #11	1	0	0
		PsycINFO	PsycARTICLES	TripDatabase**
#13.	*"fitness" AND "placebo effect"*	12	0	37
#14.	*"fitness" AND "nocebo effect"*	0	0	2

[1]*PubMed;* [2]*Cochrane;* [3]*Embase;* **Filters: TripDatabase = Primary Research;

Studies that do not meet the inclusion criteria

No placebo group and/ or no natural history/ control group

1. TODAY Study Group. (2007). Treatment options for type 2 diabetes in adolescents and youth: a study of the comparative efficacy of metformin alone or in combination with rosiglitazone or lifestyle intervention in adolescents with type 2 diabetes. Pediatric diabetes, 8(2), 74-87.
2. Tsang, T. W., Kohn, M., Chow, C. M., & Singh, M. F. (2009). A randomized controlled trial of Kung Fu training for metabolic health in overweight/obese adolescents: the" martial fitness" study. Journal of Pediatric Endocrinology and Metabolism, 22(7), 595-608.
3. Garibay-Nieto, N., Queipo-García, G., Alvarez, F., Bustos, M., Villanueva, E., Ramírez, F., ... & López-Alvarenga, J. C. (2017). Effects of conjugated linoleic acid and metformin on insulin sensitivity in obese children: Randomized clinical trial. The Journal of Clinical Endocrinology & Metabolism, 102(1), 132-140.
4. Solon, F. S., Sarol, J. N., Bernardo, A. B. I., Solon, J. A. A., Mehansho, H., Sanchez-Fermin, L. E., … Juhlin, K. D. (2003). Effect of a Multiple-Micronutrient-Fortified Fruit Powder Beverage on the Nutrition Status, Physical Fitness, and Cognitive Performance of Schoolchildren in the Philippines. Food and Nutrition Bulletin, 24(4_suppl2), S129–S140.

Figure S1. Study flow diagram.

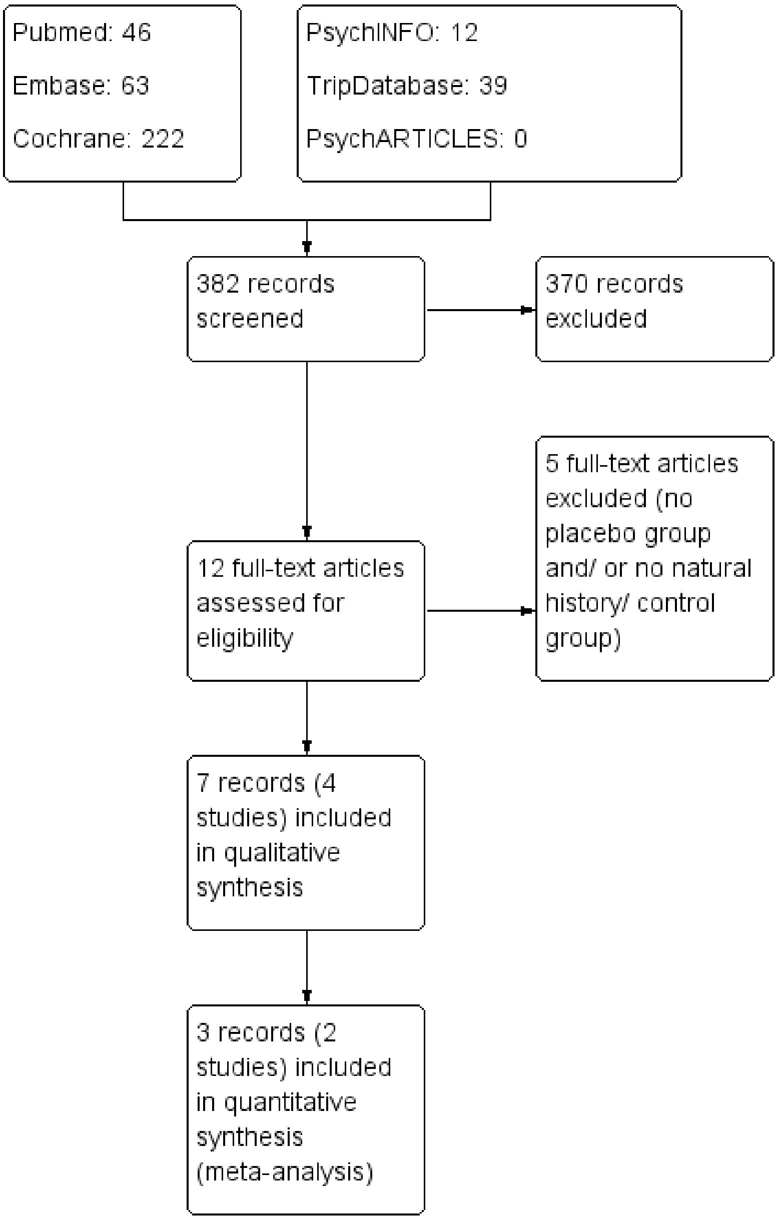

Supplement: Supplementary file 1 — Want to be fit - supplementary material [file 41366_2023_1413_MOESM1_ESM.pdf]
